# A Combined Bony and Soft Tissue, Thoracic Chance Fracture: Late Displacement following Conservative Treatment

**DOI:** 10.1155/2017/6528673

**Published:** 2017-04-10

**Authors:** James Thomas Bourne, Alexander David Laing Baker, Manoj Khatri

**Affiliations:** Lancashire Teaching Hospitals NHS Foundation Trust, Department of Trauma & Orthopaedic Surgery, Royal Preston Hospital, Sharoe Green Lane, Fulwood, Preston, Lancashire PR2 9HT, UK

## Abstract

We report the first case of a combined bony and soft tissue Chance fracture in the thoracic spine, with late presenting displacement following conservative management. Chance fractures are flexion-distraction injuries to the spine. They consist of disruption and longitudinal separation of the posterior elements of the vertebra, with the fracture extending through the pedicles and into the vertebral body. Both bony and soft tissue Chance fractures of the lumbar spine have been reported, as well as bony Chance fractures in the thoracic spine. This case suggests that this type of fracture is unstable and is an indication for operative management. It is also important to note that the displacement of the fracture occurred at more than eight weeks after injury, suggesting that instability may not present immediately.

## 1. Introduction

Chance fractures are flexion-distraction injuries to the spine, usually seen in individuals who have been restrained by a seatbelt during a road traffic accident (RTA) [1]. First described by Chance in 1948 [2], bony Chance fractures consist of disruption and longitudinal separation of the posterior elements of the vertebra, with the fracture extending through the pedicles and into the vertebral body [1].

Soft tissue Chance fractures have the same mechanism of injury, but affect the soft tissues rather than the bony structures. In a soft tissue Chance fracture there is rupture of the interspinous ligaments, facet joint capsules, and ligamentum flavum posteriorly and the posterior longitudinal ligament and intervertebral disc anteriorly [1]. Both bony and soft tissue Chance fractures of the lumbar spine have been widely reported, and there have also been reports of bony Chance fractures in the thoracic spine [3, 4].

We report a case of combined bony and soft tissue Chance fracture in the thoracic spine, which, to the authors' knowledge, is the first such report in the literature.

## 2. Case Report

A 27-year-old man was involved in a road traffic accident (RTA), where he was a passenger restrained with a 3-point seat belt. Initial investigation at the local Emergency Department (ED) revealed posterior rib fractures and a haemothorax, which was treated by insertion of a chest drain. Further imaging of the thorax by computed tomography (CT) and magnetic resonance imaging (MRI) revealed a posterior element fracture at the level of T8.

**Figure 1:**
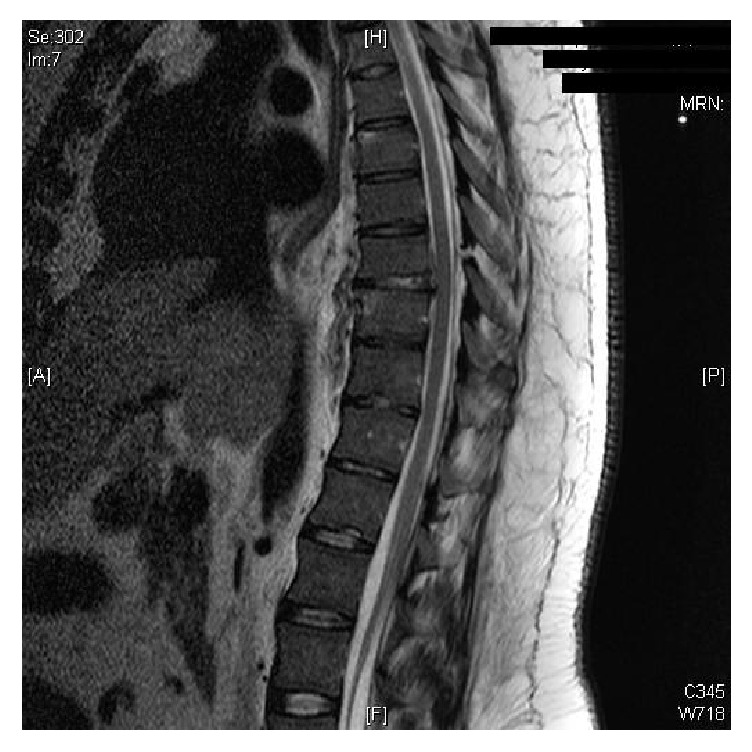
MRI of the thoracic spine on presentation to the ED, showing rupture of the ligamentum flavum at T7/8 and a hyperintense signal in the intervertebral disc at T8/9.

Four days later he was transferred to our centre for clinical review and further treatment with regard to his spinal injuries. Review of the imaging by the orthopaedic team at this time revealed a possible combined bony and soft tissue Chance fracture, with a rupture of the ligamentum flavum at T7/8 and a hyperintense signal in the intervertebral disc at T8/9 on MRI (Figure 1). The patient was counselled as to the possible unstable nature of this type of fracture and was informed of the risks and benefits of both surgical and conservative management. After long discussion with the surgical team, family members, and members of the religious community, the patient opted for conservative management.

A thoracic brace was fitted on day 7 after injury, and the patient was mobilised. Anteroposterior (AP) and lateral x-rays of the thoracic spine were taken to demonstrate the alignment of the vertebrae. The patient was discharged with paracetamol and dihydrocodeine PRN for analgesia, with follow-up planned one week later to monitor progression of the thoracic kyphosis. At the first follow-up appointment lateral X-ray of the thoracic spine showed no displacement of the vertebrae, and fortnightly follow-up was arranged thereafter.

**Figure 2:**
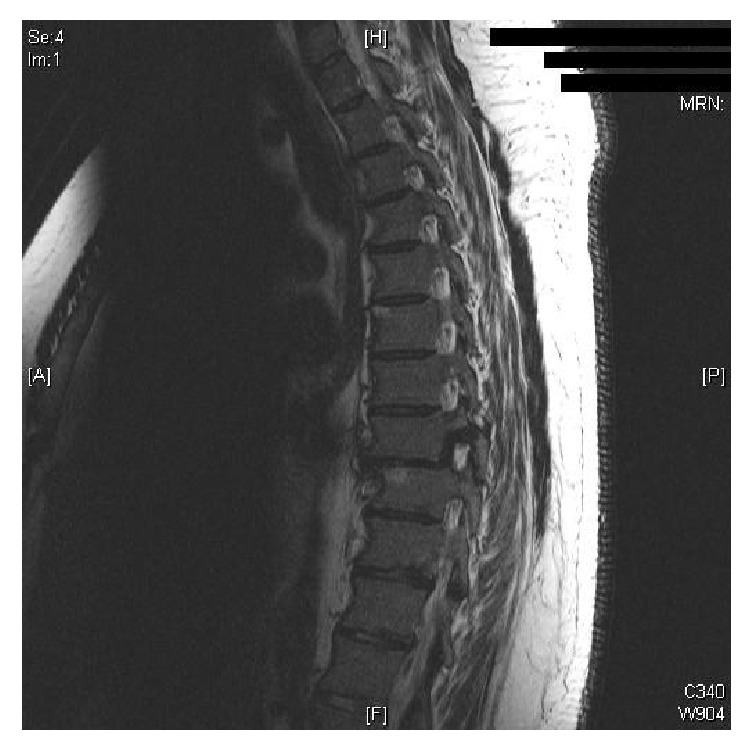
MRI of the thoracic spine at 10 weeks following initial presentation, showing a traumatic spondylolisthesis of T8 on T9.

The patient developed pleuritic chest pain four days following this first appointment and was investigated by CT pulmonary angiogram (CTPA) to exclude pulmonary embolism (PE). CTPA was negative for PE and also demonstrated that there continued to be no displacement of the thoracic spine. Further x-rays at three and five weeks after discharge also demonstrated no change to thoracic alignment. However, at ten weeks following initial presentation, the patient complained of increased thoracic back pain, and lateral X-ray of the thoracic spine suggested displacement of the vertebra at the fracture site. MRI confirmed a traumatic spondylolisthesis of T8 on T9 (Figure 2). The patient had been compliant with brace treatment and had worn the brace day and night for 6 weeks (7 weeks after injury) and then for a further 3 weeks when out of bed and mobilising.

The patient was still neurologically intact but was advised by the surgical team that the risks of continuing conservative management now greatly outweighed the risks of surgery. He underwent instrumented posterior stabilisation and fusion with allograft bone from T7 to T10 eight weeks later.

## 3. Discussion

Chance fractures are strongly associated with the use of lap-style seatbelts, with the seatbelt acting as the fulcrum about which the flexion-distraction injury occurs. As a result, the majority of Chance fractures reported in the literature occur in the lumbar or lower thoracic regions [5]. Bony Chance fractures occur in this region more frequently than soft tissue fractures, as the tensile strength of ligaments is greater than that of bone, and the bony components are more susceptible to failure [3]. Due to the mechanism by which they occur, around a third of patients with thoracolumbar Chance fractures will have a concomitant intra-abdominal injury [6].

Bony Chance fractures have also been described in the high thoracic region, in both the adult and paediatric populations [3, 4]; these are less common, due to the relative stability of the thoracic spine. However, to the authors' knowledge, this is the first report of a combined bony and soft tissue Chance fracture.

By the classification system proposed by Denis [7], Chance fractures involve disruption of both the posterior and middle columns of the spine and are therefore unstable in flexion. The instability of these fractures means they are an indication for operative treatment in adults [8]. Chance fractures have sometimes been treated conservatively using hyperextension casts, but this is not always practical due to the high number of patients with concomitant intra-abdominal injuries, in whom laparotomy will have been performed [8]. There is one report of a patient developing late complications following initial conservative management and needing subsequent operative treatment to relieve neurological symptoms [9].

**Figure 3:**
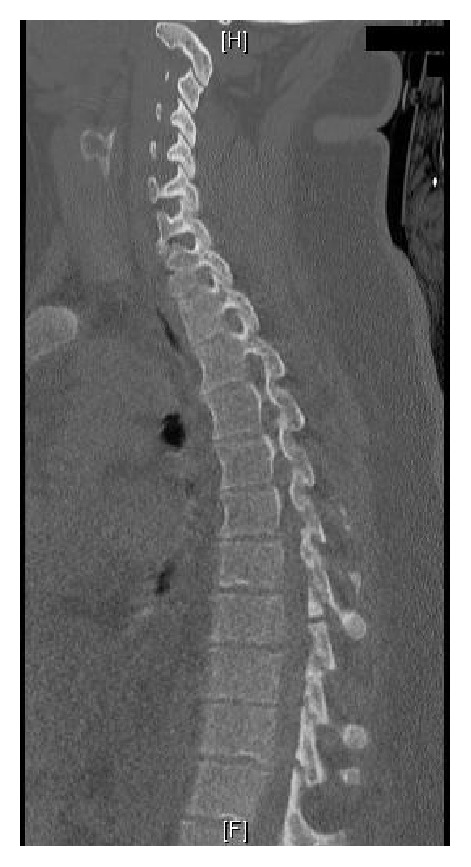
CT of the thoracic spine on presentation to the ED, showing a fracture through the lamina of T8.

The lack of reports of thoracic combined bony and soft tissue Chance fractures in the literature made it difficult to assess the stability of the fracture in this case and to advise the patient as to the relative risks and benefits of conservative and operative management. The fracture was not a classical soft tissue Chance fracture, as it disrupted the posterior elements at T7/8, and the intervertebral disc at the level below (Figure 1). There was, therefore, some bony involvement, with the fracture passing through the lamina of T8 (Figure 3). While the bony fracture was confined to the posterior element and was therefore stable, the soft tissue injuries disrupted both the posterior and middle columns of the spine.

The patient's decision to undergo conservative management in this case provides new information about the natural history of combined bony and soft tissue Chance fractures. This case suggests that this type of fracture is unstable and is an indication for operative management. It is also important to note that the displacement of the fracture occurred at more than eight weeks after injury, suggesting that instability may not present immediately.
